# Rewiring lipid metabolism to enhance immunotherapy efficacy in melanoma: a frontier in cancer treatment

**DOI:** 10.3389/fonc.2025.1519592

**Published:** 2025-05-01

**Authors:** Lihua Xiong, Jian Cheng

**Affiliations:** ^1^ Department of Dermatology, Cheng Du Xinjin District Hospital of Traditional Chinese Medicine, Chengdu, China; ^2^ Department of Chinese Medicine, Sichuan Provincial People’s Hospital, School of Medicine, University of Electronic Science and Technology of China, Chengdu, China

**Keywords:** melanoma, lipid metabolism, immunotherapy, immune checkpoint inhibitor, fatty acid oxidation (FAO)

## Abstract

Immunotherapy has transformed the landscape of melanoma treatment, offering significant extensions in survival for many patients. Despite these advancements, nearly 50% of melanoma cases remain resistant to such therapies, highlighting the need for novel approaches. Emerging research has identified lipid metabolism reprogramming as a key factor in promoting melanoma progression and resistance to immunotherapy. This reprogramming not only supports tumor growth and metastasis but also creates an immunosuppressive environment that impairs the effectiveness of treatments such as immune checkpoint inhibitors (ICIs). This review delves into the intricate relationship between lipid metabolism and immune system interactions in melanoma. We will explore how alterations in lipid metabolic pathways contribute to immune evasion and therapy resistance, emphasizing recent discoveries in this area. Additionally, we also highlights novel therapeutic strategies targeting lipid metabolism to enhance immune checkpoint inhibitor (ICI) efficacy.

## 1 Introduction

Melanoma is a malignant disease that originates from melanocytes in the skin and occurs primarily in white population. In 2021, melanoma was responsible for approximately 106,110 new cases and 7,180 deaths in the United States (1). While melanoma accounts for only 1% of skin cancers, it leads to the majority of skin cancer-related deaths due to its aggressive nature and high metastatic potential (2). This is especially critical for patients with distant metastases, whose five-year survival rate remains around 27%, even with advanced treatment options such as immune checkpoint inhibitors (ICIs) targeting PD-1 and CTLA-4 pathways (3). Nevertheless, like many chemotherapies, immunotherapy faces the inevitable challenge of clinical resistance, underscoring the importance of exploring its underlying causes to improve treatment outcomes.

The introduction of ICIs has revolutionized melanoma treatment, offering unprecedented improvements in survival for some patients. However, around half of melanoma cases exhibit primary or acquired resistance to these therapies, underscoring the urgent need to understand the underlying mechanisms of this resistance. Recent attention has shifted toward the role of metabolic reprogramming, particularly lipid metabolism, in driving melanoma progression and immune escape. This represents a critical, yet underexplored, frontier in understanding melanoma biology.

Lipid metabolism is indispensable for cellular function, serving not only as a key energy source but also as a mediator of signal transduction, membrane synthesis, and metabolic homeostasis. The regulation of lipid metabolism, including lipid uptake, synthesis, and hydrolysis, is essential for maintaining cellular homeostasis (4). However, during tumor development, cancer cells often undergo lipid metabolic reprogramming, which supports tumor growth, metastasis, and contributes to drug resistance (5). Emerging evidence points to a strong correlation between elevated lipid synthesis and melanoma aggressiveness. For example, overexpression of fatty acid synthase (FASN) has been implicated in melanoma progression, contributing to tumor growth and metastasis through its role in producing lipids that promote cell membrane synthesis and signaling molecules (6). Moreover, lipid metabolites within the tumor microenvironment (TME) actively participate in shaping immune responses. Long-chain fatty acids secreted by melanoma cells can induce dysfunction in CD8^+^ T cells, reducing their cytotoxic activity against tumor cells (7). Similarly, excess cholesterol produced by melanoma has been shown to impair CD8^+^ T cell function through mechanisms of T cell exhaustion (8). These findings emphasize that lipid metabolism reprogramming not only facilitates tumor progression but also plays a critical role in immune evasion, limiting the efficacy of immunotherapies.

Despite growing recognition of the interplay between lipid metabolism and immune evasion, there remains a significant gap in our understanding of how these metabolic alterations can be therapeutically targeted. The recent preclinical studies suggest that inhibiting lipid metabolism could enhance the response to ICIs, the mechanisms by which metabolic interventions improve immunotherapy outcomes are not fully elucidated (9). A recent study identified tectonic family member 1 (TCTN1) as a critical regulator of Fatty Acid Oxidation (FAO) in melanoma, promoting both tumor invasiveness and metastatic potential facilitating the interaction between HADHA and HADHB, subunits of the mitochondrial trifunctional protein (MTP) complex, which catalyzes FAO. This increased FAO activity fuels ATP production, enabling melanoma cells to survive under metabolic stress, evade immune surveillance, and migrate more aggressively (10). Notably, clinical data revealed that TCTN1 overexpression correlates with increased metastasis and poorer survival outcomes. Hence, there is also a need to develop more biomarkers that can predict which patients will benefit from such combination therapies, allowing for more personalized treatment strategies. This review seeks to provide a comprehensive analysis of the rewiring of lipid metabolism in melanoma and its impact on immune system interactions. By synthesizing the latest research, we will explore how lipid metabolic pathways contribute to immune evasion and therapy resistance. Additionally, we will highlight novel therapeutic strategies that target lipid metabolism to enhance immunotherapy efficacy, aiming to propose new avenues for overcoming the limitations of current treatment approaches.

## 2 Mechanisms of lipid metabolism in tumor progression

Lipids play a multifaceted role in cancer progression and metastasis, influencing various processes such as cell growth, survival, migration, and the ability to evade immune surveillance (11, 12). Tumor cells frequently reprogram lipid metabolism to support these processes, leveraging the TME for energy, structural components, and signaling molecules.

### 2.1 Lipid transporters and uptake

Tumor cells actively increase lipid uptake from the TME to meet their high metabolic demands. Lipid transporters such as CD36 and aquaporin 7 (AQP7) are upregulated, enhancing the import of fatty acids and glycerol, respectively (13). CD36 facilitates the uptake of long-chain fatty acids, which are then used to fuel energy production and biosynthetic pathways (14). This process has been linked to enhanced metastatic potential in multiple cancers, including breast and ovarian cancers (15, 16). And AQP7 favors the glycerol uptake of cancer cell, which can be used in triglyceride synthesis, contributing to lipid storage and energy production (17).

### 2.2 Lipolysis and lipid storage

In normal mammalian cells or tumor cells, fatty acids can be stored in lipid droplets or undergo lipolysis for immediate energy use. The enzyme hormone-sensitive lipase, encoded by LIPE gene, is involved in lipolysis, breaking down stored triglycerides into free fatty acids that can be used for energy production (18). This process is crucial for tumor cells to generate ATP during periods of metabolic stress (19). Lipid droplets serve as an energy reservoir, and their accumulation has been associated with increased survival under nutrient-poor conditions. For instance, in hepatocellular carcinoma (HCC), lipid droplets provide a ready supply of fatty acids for energy production when needed, giving tumor cells a survival advantage in fluctuating environments (20).

### 2.3 Fatty acid oxidation

FAO is a key metabolic pathway that cancer cells utilize to generate ATP, particularly under nutrient-limited conditions (21). By oxidizing fatty acids, tumor cells can sustain their energy needs even when glucose is scarce. The enzyme carnitine palmitoyltransferase 1 (CPT1) is crucial for transporting fatty acids into the mitochondria for oxidation, and its upregulation has been associated with increased aggressiveness and resistance to therapy in several cancers, including prostate cancer and melanoma (22, 23). For example, recent evidence suggests that lymph node metastasis require a metabolic shift towards FAO mediated by MITF, a transcription factor, in MYC^+^ melanoma. Additionally, inhibiting FAO in MYC^+^ melanoma cells could effectively reduce lymph node metastasis favored by MITF, which provided a potential therapeutic approach in melanoma dissemination (24).

### 2.4 Lipid detoxification and ferroptosis

Tumor cells also employ lipid-detoxifying enzymes to manage the buildup of toxic lipid byproducts. Aldo-keto reductase family 1 member C1 (AKR1C1) is one such enzyme that helps detoxify reactive lipid species, protecting tumor cells from lipid-induced oxidative damage (25). This detoxification mechanism is vital for maintaining cellular integrity and supporting tumor survival, particularly in the lipid-rich environment of many tumors.

Ferroptosis, a form of programmed cell death driven by iron-dependent lipid peroxidation, has emerged as a crucial metabolic vulnerability in melanoma (26). While ferroptosis can limit tumor growth, melanoma cells frequently upregulate antioxidant defense mechanisms, such as glutathione peroxidase 4 (GPX4), to escape ferroptotic cell death. GPX4 neutralizes lipid peroxides, protecting melanoma cells from oxidative damage and promoting their survival within the lipid-rich tumor microenvironment. Recent studies indicate that inducing ferroptosis in melanoma cells enhances T cell-mediated cytotoxicity and overcomes PD-1 blockade resistance. For example, inhibition of GPX4 or acyl-CoA synthetase long-chain family member 4 (ACSL4), a key enzyme that promotes lipid peroxidation, significantly enhances the efficacy of ICIs in melanoma models (27). Additionally, tumor-infiltrating Tregs have been shown to secrete, further dampening immune responses (28). Targeting ferroptosis regulators may therefore serve as a novel therapeutic approach to sensitize melanoma cells to immunotherapy.

### 2.5 *De novo* lipid synthesis

Cancer cells often exhibit increased *de novo* lipid synthesis to support rapid proliferation. FASN is a key enzyme in this pathway, producing fatty acids that are essential for membrane formation, energy storage, and signaling. Elevated FASN expression is common in several cancers, including breast and prostate cancers, and is associated with poor prognosis (29, 30). The overactivity of FASN not only facilitates tumor growth but also contributes to immune evasion by altering membrane composition and signaling pathways, ultimately creating a more favorable environment for tumor survival. For instance, recent studies have shown that small-molecule MHC-II inducers can promote immune detection and anti-cancer immunity by modulating cancer metabolism, including lipid processing pathways (31). These inducers help boost the immune system’s ability to recognize and attack tumor cells by enhancing antigen presentation, thereby shifting the metabolic balance away from tumor survival and towards immune-mediated clearance.

### 2.6 Cholesterol metabolism

Cholesterol is another important lipid that plays a role in cancer progression. Tumor cells often increase cholesterol synthesis and uptake to support membrane fluidity and the formation of lipid rafts, which are involved in cell signaling (32). Elevated cholesterol levels in the TME can also contribute to immune suppression. In colorectal cancer, increased cholesterol levels have been linked to the exhaustion of T cells, reducing their effectiveness in targeting tumor cells (33). By promoting T cell dysfunction, altered cholesterol metabolism aids tumor cells in evading the immune response.

### 2.7 Lipid signaling and the tumor microenvironment

Lipids also act as signaling molecules that influence the behavior of both tumor cells and stromal cells within the TME. Bioactive lipids such as prostaglandins, sphingolipids, and lysophosphatidic acid (LPA) play critical roles in promoting inflammation, angiogenesis, and immune modulation (34). A recent study found, in pancreatic cancer, lipid-derived signals contribute to the recruitment of immunosuppressive cells, such as myeloid-derived suppressor cells (MDSCs), which help the tumor evade immune detection and foster a pro-tumorigenic environment (35).

## 3 Rewiring of lipid metabolism in melanoma

Melanoma is not only marked by genetic and epigenetic alterations but also by profound metabolic reprogramming, particularly in lipid metabolism. This reprogramming serves as a key driver of tumor growth, survival, metastasis, and resistance to therapies, including immunotherapy. Understanding how melanoma cells alter their lipid metabolism provides insights into both the tumor’s biology and potential therapeutic interventions.

### 3.1 Overview of lipid metabolic pathways

Melanoma cells exhibit significant alterations in several lipid metabolic pathways, including FASN, β-oxidation, and cholesterol synthesis (**Table 1**). One of the most well-studied features of melanoma metabolism is the increased activity of FASN, which catalyzes *de novo* fatty acid synthesis (36). FASN is responsible for the conversion of acetyl-CoA and malonyl-CoA into long-chain fatty acids, which are essential for membrane formation, energy storage, and signaling molecule production (37). Overexpression of FASN has been strongly associated with poor prognosis in melanoma patients, as elevated lipid synthesis fuels tumor cell proliferation, survival, and immune evasion (38). For example, inhibiting FASN has been shown to reduce melanoma cell viability, sensitize tumors to ICIs, and slow tumor progression (39). Given the significant role of FASN in melanoma, it represents a promising target for combination therapies aimed at improving immunotherapy efficacy.

**Table 1:** Overview of key lipid metabolic pathways implicated in melanoma progression and immune modulation.

Lipid Metabolic Pathway	Key Regulators	Role in Melanoma Progression	Impact on Immune System
Fatty Acid Oxidation (FAO)	CPT1, TCTN1, MYC	Enhances metastatic potential and survival under metabolic stress	Impairs CD8+ T cell cytotoxicity and function
Lipid Uptake	CD36, FABP5	Increases lipid accumulation in the TME, promoting tumor progression	Drives T cell exhaustion and dysfunction
Cholesterol Metabolism	ACAT1, LXRs	Supports tumor growth and immune escape by modulating cholesterol balance	Promotes T cell exhaustion, reducing anti-tumor immunity
*De Novo* Lipid Synthesis	FASN, SCD	Sustains rapid proliferation and facilitates immune evasion	Reduces ICI efficacy by promoting an immunosuppressive TME
Lipid Detoxification	AKR1C1, GPX4	Protects tumor cells from lipid peroxidation-induced ferroptosis	Maintains suppressive function of Tregs and MDSCs

In addition to fatty acid synthesis, β-oxidation plays a critical role in melanoma cell survival, particularly under nutrient deprivation (40). The process of β-oxidation breaks down fatty acids into acetyl-CoA, which enters the tricarboxylic acid (TCA) cycle to produce ATP (41). Melanoma cells utilize this pathway to meet their high energy demands, especially in low-glucose environments typical of the TME. This metabolic flexibility allows melanoma cells to thrive in conditions that would normally be inhospitable. A recent research indicates that inhibiting FAO may impair the tumor’s ability to maintain its energy production, leading to reduced tumor survival under stress conditions (42). These findings suggest that FAO inhibitors could be an effective strategy to disrupt melanoma’s metabolic adaptability and improve outcomes in combination with ICIs.

Cholesterol metabolism is another key pathway dysregulated in melanoma. Cholesterol is integral not only to cell membrane structure but also to signaling pathways such as the PI3K/AKT and mTOR pathways, both of which are frequently activated in melanoma (43). Cholesterol has also been shown to play a role in immune suppression within the tumor microenvironment by promoting CD8^+^ T cell exhaustion, further facilitating immune evasion (8). Moreover, cholesterol metabolites, particularly 27-hydroxycholesterol, have been implicated in counteracting the effects of vemurafenib by promoting the growth of melanoma stem-like cells, thereby contributing to treatment resistance (44). Targeting cholesterol metabolism offers a promising avenue for therapeutic intervention. Inhibiting cholesterol synthesis has been shown to restore T cell function and enhance the efficacy of ICIs in preclinical melanoma models (45). These evidences position cholesterol metabolism as a critical target for overcoming immune resistance in melanoma patients.

### 3.2 Adaptation of tumor cells in the microenvironment

The TME in melanoma is a complex and heterogeneous niche, where metabolic crosstalk between tumor cells and immune cells is pivotal in determining the tumor’s fate (46). Melanoma cells actively reprogram their lipid metabolism, sculpting the TME in ways that not only suppress anti-tumor immune responses but also facilitate tumor progression. This metabolic adaptation is critical for melanoma’s survival and growth, especially in the face of therapeutic challenges.

Hypoxia, a common feature of the TME, significantly influences lipid metabolism in melanoma (47). Under low oxygen conditions, hypoxia-inducible factors (HIFs) are activated, leading to the upregulation of FASN and redirecting glycolytic intermediates toward lipid biosynthesis (48). This adaptation enables melanoma cells to maintain membrane integrity and signaling capacity, ensuring their survival despite oxygen scarcity (49). Furthermore, hypoxia-induced lipid accumulation is associated with increased levels of signaling lipids, such as prostaglandins and sphingolipids, which are known to promote tumor progression and resistance to therapy (50). And these metabolites are known to contribute to an immunosuppressive microenvironment by dampening T cell activity and promoting the recruitment of immunosuppressive cells like MDSCs (51). The presence of MDSCs is linked to poorer prognosis in melanoma, emphasizing the critical interplay between lipid metabolism and immune evasion (52). Recent research has also shown that melanoma cells grown in hypoxic conditions were found to be more resistant to chemotherapy, which was linked to an increased reliance on lipid synthesis and storage (53). This evidence highlights the critical role that lipid metabolism plays in enabling melanoma cells to thrive in oxygen-poor environments, reinforcing their ability to evade cell death and resist therapeutic interventions.

In addition to hypoxia, melanoma cells are frequently subjected to nutrient deprivation, particularly a lack of glucose and essential amino acids, due to insufficient vascularization and increased metabolic demand (54). To survive under these conditions, melanoma cells reprogram their metabolism and increasingly rely on FAO as an alternative energy source (55). FAO plays a crucial role in maintaining ATP production when glucose is scarce, allowing melanoma cells to adapt to metabolic stress. For example, a study by Li, Xiao-Xue et al. demonstrated that melanoma cells exposed to low-glucose conditions exhibited a significant increase in FAO activity, which supported their continued growth and survival (56). This shift towards FAO enables melanoma cells to break down fatty acids into acetyl-CoA, which is then fed into the tricarboxylic acid (TCA) cycle to generate ATP. Importantly, this metabolic adaptation not only helps tumor cells endure periods of metabolic stress but also contributes to their aggressiveness by fueling continuous proliferation. Moreover, the reliance on FAO has been linked to resistance to therapy. Studies have shown that inhibiting FAO in melanoma cells sensitizes them to treatments, such as chemotherapy and targeted therapies, by depriving them of a critical energy source (57, 58). In particular, targeting key enzymes involved in FAO, such as CPT1, has been shown to impair tumor growth and enhance the effectiveness of cancer treatments in preclinical models (59). Hence, FAO plays a pivotal role not only in sustaining melanoma under nutrient-limited conditions but also in promoting resistance to standard therapies.

## 4 Impact of lipid metabolism on immune cell function

**Figure 1:**
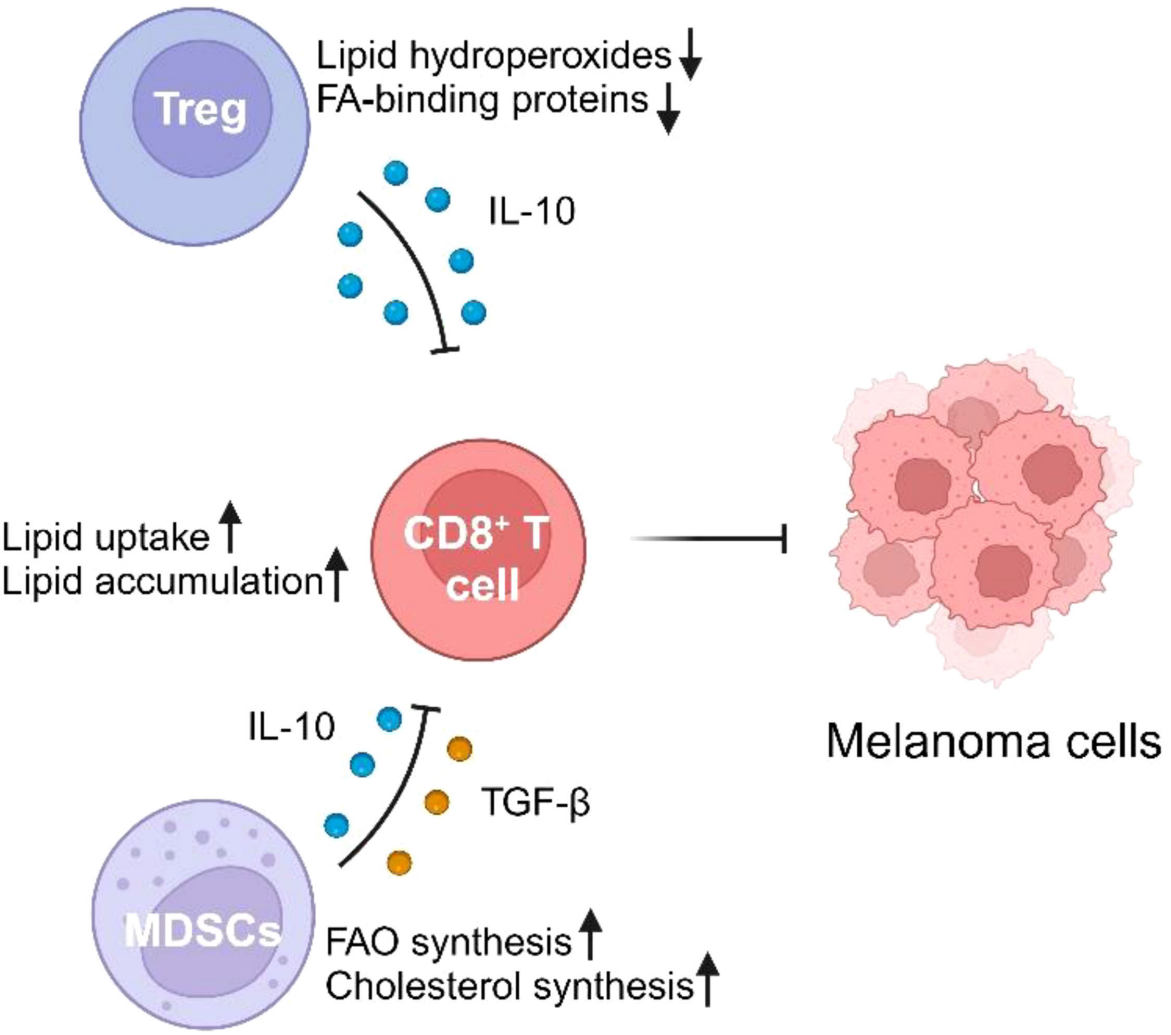
The impact of lipid metabolism on immune cells in the melanoma microenvironment. Regulatory T cells (Tregs) and myeloid-derived suppressor cells (MDSCs) negatively modulate CD8+ T cell activity through lipid metabolism-related mechanisms. Tregs reduce lipid hydroperoxides and fatty acid (FA)-binding proteins while secreting the anti-inflammatory cytokine IL-10, which inhibits CD8+ T cell activity. MDSCs promote fatty acid oxidation (FAO) and cholesterol synthesis, while releasing immunosuppressive cytokines IL-10 and TGF-β, further impairing CD8+ T cell function. CD8+ T cells, meanwhile, show increased lipid uptake and accumulation, leading to impaired anti-tumor responses against melanoma cells.

Lipid metabolism in melanoma has profound effects not only on the tumor cells themselves but also on the surrounding immune cells, significantly altering the anti-tumor immune response. Recent research highlights how metabolic reprogramming, particularly increased FAO, FASN, and cholesterol metabolism, influences the functionality of key immune cells such as cytotoxic CD8^+^ T cells, regulatory T cells (Tregs), and MDSCs (**Figure 1**). Understanding these interactions sheds light on how melanoma can evade immune surveillance and resist therapy.

### 4.1 Disruption of CD8^+^ T cell function by lipid accumulation

Recent advances in melanoma research have underscored the critical role of lipid metabolism in the dysfunction of CD8^+^ T cells within the TME. One of the key mechanisms involves the upregulation of CD36, a fatty acid transporter, which facilitates the increased uptake of fatty acids by tumor-infiltrating CD8^+^ T cells (60). This lipid overload induces oxidative stress and lipid peroxidation, leading to ferroptosis—a form of regulated cell death driven by lipid peroxides. As a result, CD8^+^ T cells experience diminished cytotoxic cytokine production and impaired antitumor capabilities, severely weakening their ability to eliminate melanoma cells (60). Importantly, studies have demonstrated that CD36 expression correlates with the upregulation of immune inhibitory receptors, such as PD-1 and TIM-3, further promoting T cell exhaustion and dysfunction (61). The metabolic stress induced by CD36-driven lipid accumulation not only triggers ferroptosis but also exacerbates T cell exhaustion, a state characterized by reduced proliferative capacity and effector function (62). This dysfunction is particularly pronounced in lipid-rich tumor environments, where the high availability of fatty acids perpetuates this deleterious cycle, effectively allowing melanoma cells to evade immune surveillance (63). Targeting these lipid metabolic pathways presents a promising therapeutic strategy. Inhibiting CD36 or blocking ferroptosis has shown significant potential in restoring CD8^+^ T cell function. Preclinical studies have demonstrated that these interventions can reinvigorate exhausted T cells and, more critically, enhance the efficacy of ICIs in melanoma (63, 64). By mitigating the lipid-induced metabolic dysfunction, such strategies offer a dual benefit: reversing immune suppression while simultaneously boosting the antitumor response.

### 4.2 Impact of lipid metabolism on Tregs, MDSCs and TAMs

Lipid metabolism plays a pivotal role in shaping the immunosuppressive function of both Tregs and MDSCs within the TME, thereby facilitating immune evasion and tumor progression.

Tregs rely heavily on lipid metabolism for their immunosuppressive function, particularly in the nutrient-deprived and immunosuppressive environment of tumors (65). FAO is essential for maintaining mitochondrial integrity and energy production in Tregs, which sustains their suppressive capacity (66). Recent studies show that the inhibition of fatty acid-binding proteins (FABPs), such as FABP5, disrupts mitochondrial integrity in Tregs, leading to the release of mitochondrial DNA and the activation of type I interferon (IFN) signaling pathways (67). This ultimately enhances IL-10 production, which bolsters the suppressive function of Tregs in the TME (67). Furthermore, lipid peroxidation poses a significant challenge to Treg function (68). Tregs are particularly susceptible to ferroptosis, a form of lipid-induced cell death. Glutathione peroxidase 4 (Gpx4) plays a key role in protecting Tregs from lipid peroxidation by neutralizing lipid hydroperoxides (69). Loss of Gpx4 in Tregs leads to impaired immune homeostasis and heightened ferroptosis, which, paradoxically, can enhance antitumor immunity by reducing Treg-mediated immune suppression (69).

MDSCs, another critical player in tumor-associated immune suppression, also depend on lipid metabolism to sustain their immunosuppressive activities. Tumor-derived factors, such as cytokines and prostaglandins, drive the differentiation and expansion of MDSCs within the TME. These myeloid cells are enriched in lipid metabolic pathways, including FAO and cholesterol synthesis, which enable them to thrive in the nutrient-poor environment of tumors and suppress the function of effector T cells (70). Moreover, increased prostaglandin E2 (PGE2) production, a common feature of lipid metabolism in tumors, has been shown to promote MDSC recruitment and enhance their suppressive function by inhibiting T cell responses and promoting the secretion of immunosuppressive cytokines such as IL-10 and TGF-β (71). Targeting lipid metabolic pathways, such as sphingosine kinase-1 (SK1) and PGE2, has been proposed as a strategy to reduce MDSC-mediated immune suppression and improve responses to immune checkpoint inhibitors (71).

Tumor-associated macrophages (TAMs) play a pivotal role in the metabolic reprogramming of the melanoma microenvironment. Recent single-cell transcriptomic analyses have identified subpopulations of lipid-associated TAMs (LA-TAMs) and angiogenic TAMs (Angio-TAMs) that exhibit distinct functions in melanoma progression. Notably, LA-TAMs1, which are enriched in lipid metabolism-related pathways, are more abundant in non-metastatic melanoma (NMM), whereas Angio-TAMs predominate in metastatic melanoma (MM) (72). These findings suggest that lipid metabolic adaptations within TAMs may shift macrophage polarization toward a more immunosuppressive and pro-metastatic phenotype, facilitating immune evasion and tumor progression. Targeting lipid metabolic pathways in TAMs could be an effective strategy to reprogram the tumor microenvironment and enhance the efficacy of immunotherapies.

### 4.3 Cholesterol metabolism and immune evasion

Cholesterol metabolism plays a crucial role in cancer progression and immune evasion within the TME. Tumor cells exhibit altered cholesterol homeostasis to support their rapid proliferation and immune escape mechanisms (73). Elevated cholesterol biosynthesis and the production of its oxidized derivatives, known as oxysterols, contribute significantly to immunosuppressive conditions within tumors. In melanoma, oxysterols such as 27-hydroxycholesterol (27-HC) activate liver X receptors (LXRs) on immune cells, leading to the recruitment of MDSCs and neutrophils into the TME (74). This recruitment suppresses effective anti-tumor immune responses, creating an environment conducive to tumor survival. Additionally, oxysterols inhibit dendritic cell migration by downregulating CCR7 expression, preventing these antigen-presenting cells from effectively initiating cytotoxic T cell responses (75). Cholesterol accumulation in melanoma also contributes to T cell exhaustion, a key factor in immune evasion. Increased cholesterol levels within CD8^+^ T cells induce mitochondrial dysfunction and upregulate inhibitory receptors such as PD-1 and TIM-3, reducing the cytotoxic function of these cells. This exhaustion severely limits the effectiveness of immune checkpoint blockade therapies (76).

## 5 Synergistic effects of targeting lipid metabolism and immunotherapy

Recent studies have demonstrated that targeting lipid metabolism can significantly enhance the efficacy of immunotherapy in melanoma (6). By manipulating lipid metabolic pathways, it is possible to improve tumor immunogenicity, thereby boosting the immune response. One notable approach involves the use of *in situ* nanovaccines, such as TPOP, which are designed to inhibit *de novo* fatty acid synthesis in tumor-infiltrating dendritic cells (TIDCs) (77, 78). TPOP nanovaccines can capture tumor antigens and enhance TIDC maturation, thereby improving their ability to cross-present antigens and activate T cells more effectively. This process ultimately results in enhanced anti-tumor immune activity, especially when combined with immune checkpoint inhibitors (77).In addition, therapies that target lipid metabolism, such as the inhibition of FASN, have been shown to increase immune checkpoint inhibitor efficacy in melanoma by altering the TME and promoting tumor immunogenicity (38, 79). For instance, FASN inhibition leads to reduced levels of immunosuppressive lipids within the tumor, enhancing the recruitment and activity of effector T cells. Proteomic analyses have revealed that high mitochondrial lipid metabolism is associated with enhanced antigen presentation and increased sensitivity to T cell-mediated killing (80). Specifically, increased mitochondrial activity leads to improved peptide processing and presentation via MHC class I molecules, which boosts T cell recognition and tumor elimination (81). This suggests that reprogramming lipid metabolism in tumors could synergize with immunotherapies like PD-1/PD-L1 inhibitors, thereby improving treatment outcomes.

Moreover, oncolytic virus-mediated approaches have been developed to potentiate the efficacy of CAR T-cell therapies against solid tumors, including melanoma (78). Oncolytic viruses can selectively infect tumor cells, causing direct oncolysis while also releasing tumor-associated antigens, which boosts immune activation (82). By using oncolytic viruses to stimulate native T-cell receptors, the expansion, activation, and antitumor function of dual-specific CAR T cells can be enhanced (78). This combined effect has shown promise in preclinical melanoma models, leading to improved tumor control and prolonged survival.

## 6 Personalized melanoma therapies based on lipid metabolism

Personalizing melanoma therapies by targeting lipid metabolism is an emerging area of research. FASN mutations have been identified as potential biomarkers for predicting the response to ICIs in melanoma patients (83). These mutations are linked to a favorable immune microenvironment and an improved prognosis for patients undergoing ICI therapy. For example, patients with high FASN expression have shown increased sensitivity to anti-PD-1 therapy, suggesting that FASN could be used as a predictive biomarker for selecting patients most likely to benefit from ICIs (38). Additionally, studies have found that inhibiting stearoyl-CoA desaturase (SCD), another key enzyme in lipid metabolism, can enhance T cell activity and improve the efficacy of ICIs (83). Such findings highlight the importance of patient-specific metabolic profiles in tailoring effective treatments. Furthermore, elevated expression of mitochondrial lipid metabolism genes has also been associated with enhanced antigen presentation and increased response to T cell-mediated killing, making mitochondrial pathways another target for personalized interventions (84).

The development of personalized vaccines that manipulate lipid metabolism within the tumor microenvironment has also shown promise (85, 86). For example, recent advances in genome engineering, such as using CRISPR to modify T-cell receptors for targeting specific lipid metabolism pathways, have shown potential to create tailored therapies for melanoma patients Additionally, targeting CAR T-cells specifically engineered to address lipid metabolic vulnerabilities within the tumor has demonstrated promising preclinical efficacy (87). Another personalized approach involves the modulation of signaling pathways, such as PPAR and glutamine metabolism, which can vary significantly between individuals, allowing for customized therapeutic regimens that maximize efficacy while minimizing side effects (86). These innovative strategies hold the potential to create individualized treatments based on the unique metabolic characteristics of a patient’s tumor.

Artificial intelligence (AI) has been increasingly applied to integrate multi-omics data, including lipidomic, genomic, and transcriptomic profiles, to predict patient responses to immunotherapy (88). AI-based models can identify complex biomarkers that integrate lipid metabolism and immune response, thereby aiding in the development of personalized treatment plans for melanoma patients (89). For instance, AI-driven analysis has been used to identify lipid metabolism-related gene signatures that correlate with improved responses to immune checkpoint blockade, providing a powerful tool for patient stratification (90). Furthermore, AI combined with radiomics has been successfully utilized to analyze imaging data such as CT and PET scans, predicting patient-specific responses to immunotherapy. By extracting imaging features that correlate with lipid metabolism, radiomics can create imaging phenotypes, which help in refining treatment strategies for individual patients (91). Machine learning algorithms have also been employed to analyze lipidomic data and predict metabolic vulnerabilities in tumors that could be targeted for therapy, thereby enabling more precise intervention strategies (92).

## 7 Future directions and clinical challenges

While combining lipid metabolism targeting with immunotherapy holds great promise, several challenges remain to be addressed. A critical issue is the heterogeneity of lipid metabolism across different melanoma patients and tumor subtypes, which complicates the standardization of treatment protocols and highlights the important of personalized therapies (93). More research is needed to understand the diverse lipid metabolic pathways and their interactions with immune responses across patient populations. Recent studies suggest that a more tailored approach, such as using advanced sequencing technologies to identify lipid-related genetic variations, could be crucial for effectively targeting these pathways in a personalized manner (94).

The translation of preclinical findings to clinical settings presents another major challenge. Although promising results have been seen in animal models, including the use of IL-10-expressing CAR T cells to overcome T cell dysfunction in solid tumors like melanoma, the efficacy of these approaches needs to be validated in human trials (79). The safety of manipulating lipid metabolism must also be rigorously evaluated, as these pathways are crucial for both tumor cells and normal cellular function. Clinical trials employing advanced imaging techniques such as radiomics, combined with AI, have shown promise in predicting treatment responses based on metabolic phenotypes, offering a way to better stratify patients for appropriate interventions.

Future research should also focus on optimizing the combination of metabolic reprogramming with established therapies, such as ICIs and CAR T-cell therapies. For instance, incorporating oncolytic viruses to boost CAR T-cell efficacy through metabolic modulation could enhance treatment durability and overcome resistance (95). Additionally, a focus on the interplay between lipid metabolism and the tumor microenvironment, specifically in nutrient-limited and hypoxic conditions, could reveal new therapeutic targets that enhance immune efficacy and prevent immune escape. Clinical trials utilizing AI to stratify patients based on metabolic and immunologic biomarkers could pave the way for more effective, individualized interventions.

## 8 Conclusion

Lipid metabolism plays a pivotal role in melanoma progression, immune evasion, and therapeutic resistance. The intricate interplay between lipid metabolism and the immune system has significant implications for developing new therapies, particularly in combination with ICIs. Advances in multi-omics and spatial transcriptomics will continue to drive the discovery of novel therapeutic targets, while personalized metabolic profiling will help tailor treatments to individual patients. Future research should focus on overcoming challenges such as toxicity and drug resistance by developing safer delivery methods and combination therapies. By integrating metabolic reprogramming with immune checkpoint inhibition, we can enhance the effectiveness of existing therapies and pave the way for more durable, long-lasting melanoma treatments.
